# Fpr1p mediates the synergistic effect of rapamycin or tacrolimus with caspofungin in *Clavispora lusitaniae in vitro*

**DOI:** 10.1093/jacamr/dlag091

**Published:** 2026-05-22

**Authors:** Maxime Lefranc, Isabelle Accoceberry, Valérie Fitton-Ouhabi, Nicolas Biteau, Thierry Noël

**Affiliations:** Univ. Bordeaux, CNRS, MFP, UMR 5234, Bordeaux F-33000, France; CHU de Bordeaux, UMR 5234, Bordeaux F-33000, France; Univ. Bordeaux, CNRS, MFP, UMR 5234, Bordeaux F-33000, France; CHU de Bordeaux, UMR 5234, Bordeaux F-33000, France; Univ. Bordeaux, CNRS, MFP, UMR 5234, Bordeaux F-33000, France; Univ. Bordeaux, CNRS, MFP, UMR 5234, Bordeaux F-33000, France; Univ. Bordeaux, CNRS, MFP, UMR 5234, Bordeaux F-33000, France

## Abstract

**Objectives:**

Caspofungin is an echinocandin antifungal agent that inhibits glucan synthesis, an essential component of the fungal cell wall. Rapamycin and tacrolimus are immunosuppressant drugs that share the same cellular receptor, the peptidyl-prolyl isomerase Fpr1p. This study investigated the interactions between rapamycin or tacrolimus and caspofungin in inhibiting the growth of wild-type *Clavispora lusitaniae* and isogenic strains engineered to overexpress or lack the *FPR1* gene.

**Methods:**

Drug interactions were assessed using the microdilution checkerboard method in liquid medium. The results were analysed using the Fractional Inhibitory Concentration Index (FICI) and Response Surface (RS) modelling via SynergyFinder 3.0.

**Results:**

Synergy was consistently observed between caspofungin and tacrolimus, as well as between caspofungin and rapamycin, in the *C. lusitaniae* wild-type strain across all tested combinations and analytical models. Deletion of *FPR1* suppressed synergy, although a weak effect between rapamycin and caspofungin persisted in the Fpr1p-deficient strain, suggesting a small Fpr1p-independent contribution of rapamycin.

**Conclusions:**

The synergistic effect of caspofungin with tacrolimus and rapamycin in *C. lusitaniae* is largely dependent on Fpr1p. The observed effects are likely mediated through inhibition of the calcineurin and TOR pathways, respectively.

## Introduction

Invasive candidiasis remains a major clinical concern, with a shift from *Candida albicans* toward non-*albicans* species that increasingly exhibit antifungal resistance.^1,2^  *Nakaseomyces glabratus* displays reduced susceptibility to azoles and emerging echinocandin resistance,^3^ while fluconazole resistance has expanded notably among clonal *Candida parapsilosis* isolates in hospital settings.^4–6^  *Candidozyma auris*, a multidrug-resistant pathogen capable of persistent colonization and rapid nosocomial transmission, is now recognized as a critical priority pathogen.^7–9^ Echinocandins remain the cornerstone of therapy, targeting β-(1,3)-glucan synthase and disrupting fungal cell wall integrity.^10^ However, the increasing emergence of resistance highlights the need to explore alternative strategies, including novel antifungal agents and combination approaches to enhance or restore antifungal susceptibility.

The main mechanism of echinocandin resistance involves non-synonymous mutations in the glucan synthase-encoding *FKS* genes, which decrease the sensitivity of the enzyme to the drug.^11^ In addition, regulators of cell stress responses such as the calmodulin-calcineurin (CaM/CaL) may contribute to the development and maintenance of echinocandin tolerance,^12–15^ resulting in increased chitin synthesis^16^ and cell wall remodelling.^17^ In *C. albicans*, tolerance and resistance to echinocandins involve the Hsp90 chaperone and its client protein, calcineurin.^18^ In *Clavispora* (*Candida*) *lusitaniae,*^19^ the calcineurin inhibitor tacrolimus (FK506) increases susceptibility to caspofungin in strains both susceptible and resistant to echinocandins, particularly in strains harbouring an Fks1 amino acid substitution at the equivalent S645 position relative to *C. albicans.*^20^ The cellular receptor of tacrolimus is Fpr1p (FK506-sensitive proline rotamase 1), a peptidyl-prolyl isomerase. Fpr1p also binds rapamycin, a natural product of *Streptomyces hygroscopicus*, which was first described as an antifungal agent that inhibits *C. albicans* growth^21^ but was later shown to have strong immunosuppressive activity.^22^ The cellular target of rapamycin is TOR complex 1 (TORC1), which is involved in the regulation of many cellular processes, in particular stress responses.^23,24^ We recently demonstrated a synergy between caspofungin and rapamycin in caspofungin-susceptible and caspofungin-resistant *C. albicans and C. lusitaniae* strains *in vitro.*^25^ Fpr1p is thus at the crossroads of the signalling routes triggered by rapamycin and tacrolimus. To gain information on the interaction between caspofungin and immunosuppressive drugs, we evaluated the *in vitro* inhibitory activity of caspofungin in combination with rapamycin or tacrolimus in *C. lusitaniae* using wild-type (WT) and isogenic mutant strains either lacking or overexpressing the *FPR1* gene. Although less frequently associated with invasive candidiasis, *C. lusitaniae* is an emerging opportunistic pathogen with documented antifungal resistance^26,27^ and phylogenetic proximity to *C. auris.*^28^ Its haploid genome, fully sequenced and annotated,^29^ and its high genetic tractability, including the possible use of genetic crosses,^30^ enable direct genotype–phenotype relationships. It is readily accessible to genome editing via genetic transformation and homologous recombination, which supports its use as a laboratory-relevant model for functional validation of clinical antifungal resistance determinants.^31–33^

## Materials and methods

### Yeast strains and culture conditions

Strains are listed in Table 1. The *C. lusitaniae* CBS 6936 strain served as the WT reference for gene cloning and antifungal susceptibility testing. The auxotrophic mutant 6936 *ura3*Δ (harbouring a 990-bp deletion in the orotidine 5′-phosphate decarboxylase gene) was utilized as the parental strain for generating *fpr1*Δ, *FPR1*C, and *FPR1*re mutants. Each construct was independently generated in three separate isolates to ensure phenotypic reproducibility. Yeasts were cultivated in liquid YPD medium (1% yeast extract, 2% peptone, and 2% dextrose) at 35°C under agitation (250 rpm). For transformation, selection was achieved on synthetic YNBS medium (0.67% yeast nitrogen base without amino acids [Difco Laboratories, Detroit, USA]; 2% glucose; 1 M sorbitol), supplemented with uracil (50 mg/L), and/or 5-fluoro-orotic acid (0.8 g/L) when required.

**Table 1.** Names, genotypes, and phenotypes of the strains of *C*. *lusitaniae* constructed and used in this study

Strain	Origin^a^	Genotype	Phenotype	Reference
CBS 6936	Citrus peel	WT	WT	(22)
6936 *ura3Δ990*	CBS 6936	*ura3Δ _[990]_*		(23)
*fpr1Δ*	6936 *ura3Δ990*	*fpr1Δ::URA3*	Fpr1p^null^	This study
*FPR1C*	6936 *ura3Δ990*	*FPR1*, *pADH-FPR1*, *URA3*	Fpr1p^oe^	This study
*FPR1re*	*fpr1Δ::URA3*	*fpr1Δ::FPR1*, *ura3Δ*	WT	This study

^oe^over-expressed.

^a^CBS, Centraal Bureau voor Schimmelcultures, renamed the Westerdijk Fungal Biodiversity Institute.

### DNA engineering, molecular assembly, and yeast transformation

Genomic DNA was extracted using the glass bead method.^34^ High-fidelity DNA polymerase Pfu (Promega, Madison, USA) was used for cloning and overlapping PCR, while GoTaq or GoTaq Long (Promega) were employed for routine screenings and targets up to 5 kb. Primers (Table 2) were synthesized by Eurofins MWG Operon (Ebersberg, Germany).

**Table 2.** Oligonucleotides used in this study

Usage/Destination	Primer name	Sequence 5’-3’^a^
Cassette construction for inserting *URA3* in the *FPR1* gene of *C. lusitaniae*	p*Clus FPR1*-fam	cggtacccggggatcGATTTCTTCTGTTTCCCTAATG
	*Clus FPR1*-ram2	tctgccatcaacgggATTTGAGTAGTTTGAGGAGCAG
	F*URA3*	CCCGTTGATGGCAGAGTTGG
	*URA3*-3UTR-R	CGAGATGACATTCTCCATTC
	*Clus FPR1*-fav	gagaatgtcatctcgGTGCATTTTGTTCTAGCTAATG
	p*Clus FPR1*-rav	cgactctagaggatcGTGAAGTTGAAATACACCATTG
Amplifying cassette for inserting *URA3* in the *FPR1* gene	*Clus FPR1*-fam2	GATTTCTTCTGTTTCCCTAATG
	*Clus FPR1*-rav	GTGAAGTTGAAATACACCATTG
Screening correct insertion of *URA3* at the targeted *FPR1* locus	*Clus FPR1* Fext	CTAAAGGAGCTAATGCACAGC
	*Clus FPR1* Rext	GACACCATCAATTACTATGAGC
Cassette construction for inserting a copy of *FPR1* under the promotor	p*Clus-*p*ADH1*-F1	ctcccggccgccatgTGCGATCTTGGTCAGGTTGTGTTC
*ADH* in the 5′ UTR *FPR1* region of *C. lusitaniae*	*Clus-FPR1-*p*ADH1*-R1	ttgaggagcagacatTCTTTGTTTTTTTCTAAGGGGATTC
	*Clus-FPR1-*ATG	ATGTCTGCTCCTCAAACTACTC
	p*Clus-FPR1-*R1	cccgcggccgccatgGTGAAGTTGAAATACACCATTG
Amplifying cassette for inserting a copy of *FPR1* under the promoter *ADH*	p*Clus-*p*ADH1-*F1	ctcccggccgccatgTGCGATCTTGGTCAGGTTGTGTTC
in the 5′ UTR *FPR1* region of *C. lusitaniae*	p*Clus-FPR1*-R1	cccgcggccgccatgGTGAAGTTGAAATACACCATTG
Screening the correct insertion of a copy of *FPR1* under the promoter *ADH* in	3*URA3*-F2	ATTGTTCCAGAGTTGATCCG
the 5′ UTR *FPR1* region of *C. lusitaniae*	*Clus FPR1* Fext	CTAAAGGAGCTAATGCACAGC
	*Clus FPR1* Rext	GACACCATCAATTACTATGAGC
	p*Clus-*p*ADH1-*F1	ctcccggccgccatgTGCGATCTTGGTCAGGTTGTGTTC
RT-qPCR	*ACT1* F1	CGCGCTGAAAGAGAAATCGTC
	*ACT1* R1	TGGTTTTGGTCAATACCAGCA
	*FPR1* F2	CTACACTGGAACTTTGGAAAACGG
	*FPR1* R2	AACCTGGGAAACCACTTGGACC

^a^Lowercase and uppercase letters are used for hybrid primers to distinguish the parts specific to each target DNA.

To construct DNA cassettes, the different DNA fragments to be assembled were generated via PCR using primers with 15 bp homologous overlaps at their ends. Then the fragments were recombined *in vitro* and cloned in a pUC19 plasmid or a linearized pGEMU plasmid (pGEMT harbouring the *C. lusitaniae URA3* gene) using the In-Fusion HD cloning kit (Takara Bio Europe, Saint-Germain-en-Laye, France). Recombinant plasmids were propagated in *Escherichia coli* Stellar cells (Takara Bio Europe) and selected on LB agar supplemented with 50 mg/L ampicillin. DNA cassettes were PCR-amplified from recombinant plasmids using the relevant primers, purified on QIAquick columns (Qiagen, Hilden, Germany), and used for yeast transformation. Auxotrophic strains were transformed by electroporation as previously described.^32^ Prototrophic transformants were selected on YNBS agar after 3 days at 35°C.

### RT-qPCR

Total RNA was extracted using a guanidinium thiocyanate-phenol-chloroform method.^35^ Briefly, yeast strains were grown to the stationary phase and adjusted to a density of 5 × 10^8^ cells. Two independent biological replicates were prepared by resuspending cells in 3 mL of RPMI 1640 medium (Sigma-Aldrich, Saint-Louis, USA), followed by incubation for 3 h at 30°C, 215 rpm. Cells were harvested, resuspended in TRI Reagent (Molecular Research Center, Inc., Montgomery, USA), and disrupted with acid-washed glass beads using a Precellys 24 tissue homogeniser (2 × 40 s, 6800 rpm, 4°C). The aqueous phase was successively extracted with 5:1 (vol/vol) acidic phenol-chloroform (pH 5.1) and 24:1 (vol/vol) chloroform-isoamyl alcohol, then precipitated with isopropanol. Total RNA was solubilized in RNase-free water and quantified using an Agilent RNA 6000 Nano chip.

Gene expression was determined via RT-qPCR using a one-step real-time PCR system (GoTaq 1-Step RT-qPCR; Promega) and the CFX96 real-time PCR detection system (Bio-Rad Laboratories, Hercules, USA). Fold changes in gene expression relative to that of the control WT strain 6936 were determined from *ACT1*-normalized expression levels using the 2-ΔΔCt method.^36^ Primers are listed in Table 2.

### *In vitro* susceptibility testing

Stock solutions (10 mg/mL) of caspofungin, rapamycin, and tacrolimus (Euromedex, Souffelweyersheim France) were prepared in DMSO and stored at −20°C.

Microdilution assays were performed in RPMI 1640 buffered to pH 7.0 with 0.165 M 3-(N-morpholino) propanesulfonic acid (MOPS) (Euromedex) according to CLSI M27 guidelines,^37^ with specific modifications adapted for synergy modelling. Yeast inocula were standardized to a final concentration of 1 × 10^3^ cells/mL. Microtiter plates were incubated for 48 h at 35°C. Growth was quantified spectrophotometrically at 450 nm using a Fluostar Optima reader (BMG Labtech Ortenberg, Germany), as previously described.^25^ Absorbance values were subsequently converted into growth percentages to serve as input for response surface (RS) analysis.

### Minimal inhibitory concentration (MIC) determination and interaction analysis

Caspofungin (0.0625 to 4 mg/L) was tested in combination with rapamycin or tacrolimus (0.01 to 10 mg/L) using a checkerboard microdilution assay, in which 2-fold serial dilutions of each compound were combined in a two-dimensional matrix in 96-well plates, allowing the evaluation of all concentration combinations. Given that tacrolimus is not an antifungal agent and that the antifungal activity of rapamycin is not standardized, MICs were uniformly defined at 90% growth inhibition to ensure consistency across conditions. Off-scale MICs were assigned to the next higher 2-fold dilution for analysis.

Drug interactions between caspofungin and rapamycin or tacrolimus were analysed using both the Fractional Inhibitory Concentration Index (FICI) and RS modelling via SynergyFinder 3.0. FICIs were calculated from checkerboard assays and interpreted according to Journal of Antimicrobial Chemotherapy guidelines as synergistic (FICI ≤0.5), indifferent (0.5  < FICI ≤4), or antagonistic (FICI >4). FICI interpretations remained consistent across replicates.

To ensure a robust assessment, interaction landscapes were analysed using SynergyFinder 3.0,^38^ which evaluates synergy through four widely used models: Highest Single Agent (HSA), Loewe additivity, Bliss independence, and Zero Interaction Potency (ZIP).

The HSA model defines the expected combination effect as the maximum response of either drug at a given concentration.^39^ The Loewe additivity model calculates the expected response as if both drugs are the same.^40^ The Bliss independence model predicts the expected effect based on the statistical probability of independent events, assuming the drugs act through different mechanisms.^41^ ZIP evaluates the interaction by assuming that neither drug affects the potency of the other.^42^

For each model, synergy scores were computed across the full concentration matrix from mean growth values of three independent experiments (biological replicates, variability ≤10%). According to the system’s criteria, drug combinations were classified as antagonistic, additive, or synergistic when the synergy score was ≤−10, between −10 and 10, or >10, respectively.^38^

Statistical significance was determined against a null hypothesis (score = 0) using SynergyFinder’s non-parametric resampling framework (permutation/bootstrap), with a significance threshold of *P* < 0.05.

## Results

### Rapamycin shows Fpr1p-dependent antifungal activity in *C*. *lusitaniae* 6936 *in vitro*

**Figure 1.**
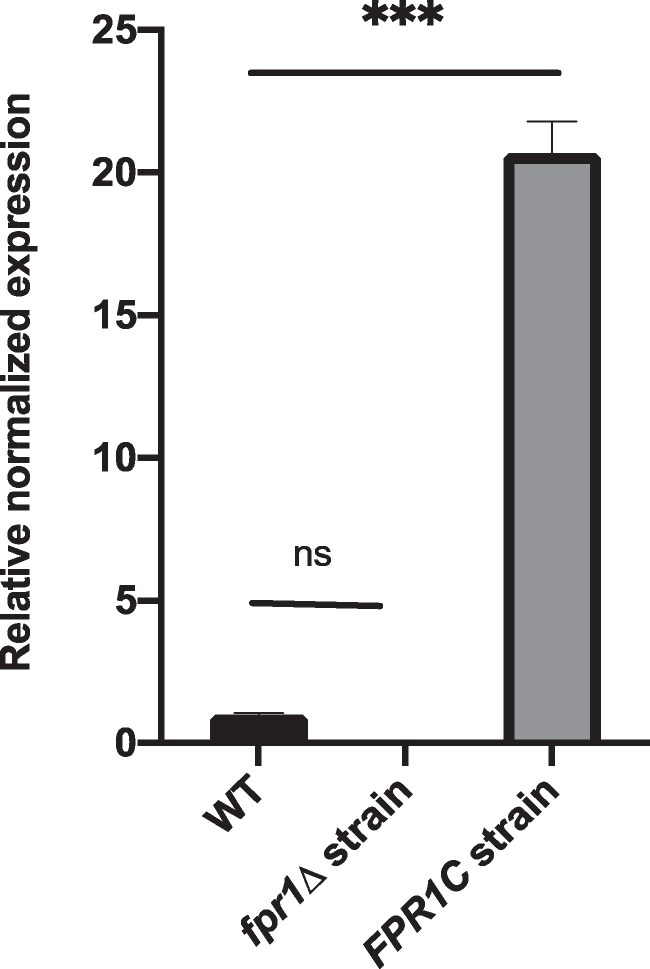
Quantitative transcriptional analysis of the *FPR1* gene in *C*. *lusitaniae* WT, *fpr1Δ*, and *FPR1c* strains. Error bars indicate standard errors of the mean (SEMs). Differences in gene expression relative to the WT were analysed using a *t*-test. ns: not significant; ***P < 0.001.

To investigate the role of the peptidyl-prolyl isomerase Fpr1p during interactions between immunosuppressive drugs and caspofungin in *C*. *lusitaniae*, we first constructed loss- and gain-of-function mutant strains in the genetic background of the 6936 strain. After verifying the correct molecular organization of the mutants via PCR, RT-qPCR was performed to verify the expression of the *FPR1* gene in the *fpr1*Δ and *FPR1*C strains compared to the 6936 WT strain. As expected, *FPR1* expression was absent in the *fpr1Δ* mutant. In contrast, *FPR1* expression in the *FPR1C* mutant was approximately 20-fold higher than in the WT strain, driven by transcription of an additional *FPR1* copy under the alcohol dehydrogenase promoter (*ADH1*) (Figure 1 and Figure S1 (available as Supplementary data at *JAC-AMR* Online)). MICs of rapamycin, tacrolimus, and caspofungin were determined for the WT strain and the mutants *fpr1Δ*, *FPR1C*, and *FPR1re*. The *FPR1re* strain carries a reconstructed *FPR1* locus generated by reintroducing a WT *FPR1* allele into the *fpr1Δ* mutant (Table 3). All strains displayed similar growth rates in drug-free medium.

**Table 3.** MICs for rapamycin, tacrolimus, and caspofungin in wild-type and *FPR1* mutant strains of *C*. *lusitaniae*

Strain	MIC rapamycin (mg/L)	MIC tacrolimus (mg/L)	MIC caspofungin (mg/L)
6936 WT	10	>320	1
*fpr1Δ*	> 320	>320	1
*FPR1C*	0.16	>320	1
*FPR1re*	10	>320	1

The MICs for each compound in each strain remained within ±2 twofold dilutions across all experiments.

The MIC of rapamycin for the 6936 WT strain of *C*. *lusitaniae* was 10 mg/L. In the *fpr1*Δ mutant, the MIC was >320 mg/L, representing more than a 32-fold increase. In the *FPR1C* mutant, the MIC was 0.16 mg/L, approximately 65-fold lower than the WT. Deletion of *FPR1* therefore conferred a high level of resistance to rapamycin in *C. lusitaniae*, whereas the introduction of an additional copy of *FPR1* under the *ADH1* promoter markedly increased susceptibility. Reintroduction of a single *FPR1* allele expressed under its native promoter into the *fpr1Δ* background restored the MIC to WT levels (10 mg/L; Table 3). These findings show that the expression level of *FPR1* directly influences the susceptibility of *C*. *lusitaniae* to rapamycin.

In the *C*. *lusitaniae* WT strain, the MIC of tacrolimus was not reached, even at the highest concentration tested (MIC >320 mg/L). This was independent of *FPR1* expression levels, as MICs were >320 mg/L for both the *fpr1*Δ (no expression) and *FPR1*C (overexpression) strains (Table 3). In these experiments, no antifungal activity of tacrolimus was observed against *C*. *lusitaniae*.

Concerning caspofungin, all of the *C*. *lusitaniae* strains exhibited an MIC of 1 mg/L, regardless of *FPR1* expression levels.

### The synergistic effect of caspofungin and rapamycin in inhibiting *C*. *lusitaniae* growth is largely, but not completely, dependent on *FPR1*

**Figure 2.**
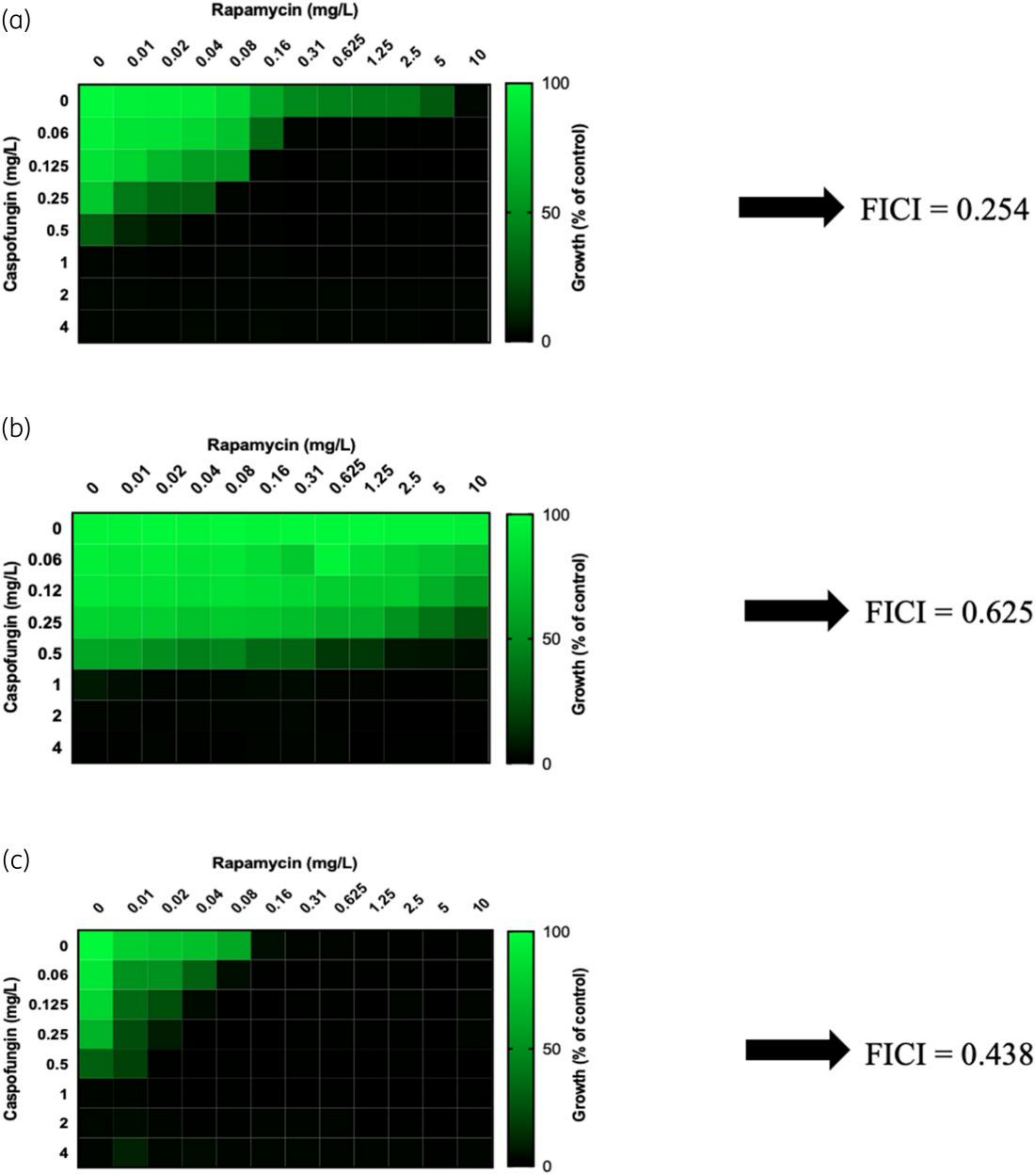
Interaction between rapamycin and caspofungin against *C*. *lusitaniae* 6936 WT (panel a), *fpr1Δ* (panel b), and *FPR1C* (panel c). Each panel presents the percentage of growth and the fractional inhibitory concentration index (FICI).

Checkerboard assays showed that the combination of caspofungin and rapamycin yielded an FICI of 0.254 for the WT strain, indicating a synergistic effect (Figure 2a and Table 4). For this strain, rapamycin at 0.31 mg/L (32-fold below its MIC) reduced the caspofungin MIC 16-fold, from 1 to 0.06 mg/L. For *fpr1*Δ, the FICI was 0.625, indicating that the combination of caspofungin and rapamycin was indifferent (Figure 2b and Table 4). The switch from synergy to indifference can be explained by the fact that deletion of *FPR1* rendered the *fpr1*Δ strain resistant to rapamycin at concentrations higher than the maximum used in the checkerboard experiments. The synergistic effect of caspofungin and rapamycin was restored in the *FPR1*C strain (FICI = 0.438, Figure 2c and Table 4). Overall, 50% growth inhibition was observed for *FPR1*C in the presence of the lowest concentrations of rapamycin (0.01 mg/L, i.e. 16-fold lower than its MIC) and caspofungin (0.06 mg/L, i.e. 16-fold lower than its MIC) tested.

**Table 4.** *In vitro* interaction between caspofungin and rapamycin against *C. lusitaniae* strains

Strains	Chequerboard MICs (mg/L)			FICI		Synergy scores^a^			
	Caspofungin alone	Rapamycin alone	Caspofungin and rapamycin in combination	Value	INT	HSA	Loewe	Bliss	ZIP
*C. lusitaniae 6936*	1	10	0.0625/0.31	0.254	SYN	20.13	16.30	15.35	15.14
*C. lusitaniae fpr1Δ*	1	>10	0.5/2.5	0.625	IND	10.51	11.11	10.51	10.55
*C. lusitaniae FPR1C*	1	0.16	0.125/0.04	0.438	SYN	10.89	7.77	7.53	8.15

*n* = 3.

IND, indifference; INT, interpretation; SYN, synergistic.

^a^For all synergy scores: *P* < 0.0001.

RS analysis of the results of the checkerboard assays with the four models (HSA, Loewe, Bliss, and ZIP) confirmed the synergistic effect of caspofungin and rapamycin for the WT strain (Table 4 and Figure S2). The mean HSA, Loewe, Bliss, and ZIP synergy scores were statistically significantly synergistic at 20.13, 16.3, 15.35, and 15.14, respectively (*P* < 0.0001) (Table 4 and Figure S2a–d). In contrast to the results obtained in the FIC analysis, for the *fpr1Δ* mutant, there was a synergistic effect between caspofungin and rapamycin, with synergy scores of 10.51, 11.11, 10.51, and 10.55 obtained with the HSA, Loewe, Bliss, and ZIP models, respectively (Table 4 and Figure S2e–h). For the *FPR1*C mutant, FICI and RS analysis yielded different interpretations. The Loewe, Bliss, and ZIP models indicated an additive effect of caspofungin and rapamycin with synergy scores of 7.77, 7.53, and 8.15, respectively (*P* < 0.0001). The HSA model interpreted the interaction as synergistic for the *FPR1C* mutant, with a synergy score slightly above 10 (10.89) (Table 4 and Figure S2i–l).

Taken together, these results highlight the involvement of Fpr1p in the synergistic growth inhibition of *C*. *lusitaniae* by caspofungin and rapamycin. Although the antifungal activity of rapamycin alone strictly depends on its interaction with Fpr1p, a synergistic effect, albeit weaker than that observed in the WT strain, is still detectable between caspofungin and rapamycin in the *fpr1*Δ mutant. This observation suggests that, in *C*. *lusitaniae*, the synergistic inhibition of growth by caspofungin and rapamycin may be at least partially independent of Fpr1p.

### The synergistic effect of caspofungin and tacrolimus is strictly dependent on *FPR1*

Checkerboard assays showed that caspofungin had a synergistic effect with tacrolimus to inhibit the growth of the *C*. *lusitaniae* WT strain. This effect yielded an FICI of 0.466 and high synergy scores (20.99, 19.93, 20.33, and 20.5 for the HSA, Loewe, Bliss, and ZIP models, respectively) (Table 5 and Figure S3). Deleting *FPR1* abolished the synergy between caspofungin and tacrolimus. In the *fpr1Δ* strain, the FICI was 0.835, classifying the interaction as indifferent according to this model. RS analysis confirmed this conclusion with synergy scores under the threshold of 10 (7.41, 6.66, 7.02, 7.3 for HSA, Loewe, Bliss, and ZIP, respectively). Overexpression of the *FPR1* gene in the strain *FPR1*C did not reduce or enhance the synergistic effect between caspofungin and tacrolimus, with FICI (FICI = 0.430) and synergy scores (18.52, 18.87, 18.15, and 18.36 for the HSA, Loewe, Bliss, and ZIP models, respectively) being similar to those observed for the WT strain.

**Table 5.** *In vitro* interaction between caspofungin and tacrolimus against *C. lusitaniae* strains

Strains	Chequerboard MICs (mg/L)			FICI		Synergy scores^a^			
	Caspofungin alone	Tacrolimus alone	Caspofungin and tacrolimus in combination	Value	INT	HSA	Loewe	Bliss	ZIP
*C. lusitaniae 6936*	1	>10	0.25/2.5	0.466	SYN	20.99	19.93	20.33	20.50
*C. lusitaniae fpr1Δ*	1	>10	0.5/0.01	0.835	IND	7.41	6.66	7.02	7.30
*C. lusitaniae FPR1C*	1	>10	0.25/0.32	0.430	SYN	18.52	18.87	18.15	18.36

*n* = 3.

IND, indifference; INT, interpretation; SYN, synergistic.

^a^for all synergy scores: *P* < 0.0001.

## Discussion

We investigated the interaction between caspofungin and the Fpr1p-binding drugs rapamycin and tacrolimus using loss- and gain-of-function *FPR1* mutants in the opportunistic pathogenic yeast *C*. *lusitaniae*. Interactions were assessed using the checkerboard method in 96-well plates, which allow us to test several tens of different concentrations in combination. This approach, however, has limitations in interpreting antifungal combinations because a consensus framework for defining drug synergy is lacking.^43^ Accordingly, in addition to the conventional FICI analysis, we also used RS analysis with different mathematical models, all with a strict synergy score threshold of 10, as previously recommended.^38^ Divergent interpretations of certain interactions were observed between the two methods. These differences arose because the FICI method is endpoint-dependent, considering only interactions at the MIC, whereas the RS analysis incorporates all interactions across the 96 wells of the plate. Another source of variation stems from the concentration ranges tested relative to the yeast genotype. For example, although constructed in the same genetic background, the loss-of-function *fpr1Δ* mutant is resistant to rapamycin, while the gain-of-function *FPR1*C mutant is markedly more susceptible than the WT reference strain. This can lead to overestimation of the FICI in the rapamycin-resistant mutant and underestimation of the synergy score by RS analysis in the *FPR1*C mutant. These considerations justified the use of multiple analytical approaches to interpret the checkerboard experiments.

The primary physiological role of Fpr1p is to accelerate the rate-limiting step of protein folding by catalyzing *cis*-*trans* isomerization of peptidyl-prolyl bonds.^44^ This peptidyl-prolyl isomerase activity promotes isomerization of the imide bond of proline residues, stabilizing proteins and thereby enhancing folding efficiency. Yalcin *et al*.^45^ recently proposed that Fpr1p also has an anti-aging function by maintaining proteostasis, particularly through the activation of ribosomal protein genes via its peptidyl-prolyl isomerase activity. Beyond its catalytic role, Fpr1p acts as a chaperone, contributing to protein folding and preventing aggregation.^46^ This dual functionality positions Fpr1p as a critical component of the cellular protein quality-control system, where its peptidyl-prolyl isomerase activity serves as the central mechanism for ensuring proper protein conformation and mitigating misfolding-associated cellular stress.

Fpr1 protein inhibits target of rapamycin complex 1 (TORC1) and calcineurin when bound to rapamycin and tacrolimus, respectively. As already reported in other fungal species,^47^ our study confirms the predominant role of Fpr1p in controlling growth of *C*. *lusitaniae* in the presence of rapamycin, demonstrating that the antifungal effect of rapamycin is abolished when the *FPR1* gene is deleted, while susceptibility to rapamycin is increased by overexpression of *FPR1*.

The principal mechanism of action of rapamycin in yeast involves its binding to Fpr1p, after which the rapamycin-Fpr1p complex associates with TORC1 and inhibits its kinase activity. TORC1 is a central regulator of cell growth and metabolism, integrating nutrient, energy, and stress signals. Inhibition of TORC1 by rapamycin results in rapid repression of ribosomal protein gene expression, a global reduction in protein synthesis, and induction of autophagy and stress–response pathways.^23,24,48,49^ This mechanism highlights TORC1 as a major node in fungal growth regulation. As previously reported,^25^ we observed a synergistic effect between rapamycin and caspofungin in the inhibition of growth of the WT strain of *C*. *lusitaniae*. Surprisingly, in the RS analysis, this synergistic effect was also observed in the *fpr1*Δ mutant. This was unexpected because the antifungal effect of rapamycin alone was strictly dependent on Fpr1p in *C*. *lusitaniae*. This suggests a potential mode of cellular action for rapamycin independent of Fpr1. Interestingly, resistance to rapamycin occurs when the *PMR1* gene is deleted. The Pmr1 pump is involved in calcium ion transport within the Golgi apparatus.^50^ Rapamycin may therefore participate in the regulation of intracellular calcium fluxes and may interfere with the activation of certain cellular calcium-dependent pathways, such as calcineurin.

We also observed no antifungal activity of tacrolimus in *C. lusitaniae*, even when its cellular receptor Fpr1p was overexpressed, consistent with similar findings reported for *C. albicans* and *Cryptococcus neoformans.*^47,51^

Fpr1p is the first member of the FKBP family identified in *Saccharomyces cerevisiae* and is the homologue of the mammalian prolyl isomerase FKBP12. Rapamycin and tacrolimus also bind FKBP12 and are widely used to prevent organ transplant rejection and control undesired immune responses.^22,52^ Consequently, it is inconceivable that these drugs could be employed to treat opportunistic fungal infections, despite our demonstration of synergistic effects in different strains of *C. lusitaniae* and *C. albicans*, whether they are susceptible or resistant to echinocandins.^25^ Nevertheless, fewer immunosuppressive analogues of rapamycin and tacrolimus have been developed.^53,54^ Recently, Juvvadi *et al*.^54^ resolved the crystal structure of calcineurin in the presence of tacrolimus and Fpr1 protein in *Aspergillus fumigatus*. They identified a specific site on the fungal Fpr1 protein, unique to fungi, that is essential for calcineurin inhibition by tacrolimus. This discovery enabled the design of APX879, a tacrolimus analogue with an acetohydrazine substitution at position C22. APX879 exhibits reduced immunosuppressive properties compared to tacrolimus. Specifically, IL-2 production by activated T lymphocytes was increased 70-fold in the presence of APX879 compared to tacrolimus, reflecting its weak immunosuppressive activity. Also, APX879 demonstrated efficacy alone and in combination with fluconazole in an *in vivo* model of *Cryptococcus neoformans* infection, but not in models of *C. albicans* candidaemia or *A*. *fumigatus* aspergillosis. Further structural modifications of APX879 may enhance its fungal specificity.^55^ Few studies have described rapamycin analogues that retain antifungal activity while reducing immunosuppressive properties.^47,56^ Other Fpr1p-binding drugs have recently been identified, although data on their antifungal potential remain scarce.^57^ The finding that the synergistic effects between caspofungin and rapamycin or tacrolimus are mediated by Fpr1p in *C*. *lusitaniae* suggests that the underlying signalling pathways (the protein kinase pathway for caspofungin, the TOR pathway for rapamycin, and the calcineurin pathway for tacrolimus) are involved in this phenomenon and are interconnected, as suggested by several previous studies.^58–61^ These findings are consistent with our previous work, which showed that inhibition of the TOR pathway restored echinocandin susceptibility in *C. albicans* and *C. lusitaniae* strains harbouring resistance-associated *FKS1* mutations.^25^ Given that the cell wall integrity, calcineurin, and TOR pathways are central to the compensatory response to echinocandin-induced cell wall stress,^62,63^ these pathways could also modulate the response to next-generation cell wall-targeting agents such as rezafungin, ibrexafungerp, and fosmanogepix.

Overall, this study provides new insights into the molecular basis of echinocandin resistance and opens up new perspectives for the management and optimization of antifungal treatments in clinical settings. It identifies TOR-regulated signalling pathways as potential targets, as well as the use of non-immunosuppressive analogues of rapamycin for the development of rational combination therapies aimed at tackling echinocandin resistance.

## Supplementary Material

dlag091_Supplementary_Data

## Supplementary data

Figures S1 to S3 is available as Supplementary data at JAC-AMR Online.
