# Supplementary material for: Fpr1p mediates the synergistic effect of rapamycin or tacrolimus with caspofungin in Clavispora lusitaniae in vitro
Source: JAC Antimicrob Resist. 2026 May 22;8(3):dlag091. doi: 10.1093/jacamr/dlag091 (PMC13195513; doi:10.1093/jacamr/dlag091)
Supplement: dlag091_Supplementary_Data [file dlag091_supplementary_data.docx]

**Supplementary data**

Fpr1p mediates the synergistic effect of rapamycin or tacrolimus with caspofungin in *Clavispora lusitaniae* *in vitro*, by **Maxime LEFRANC et al.**


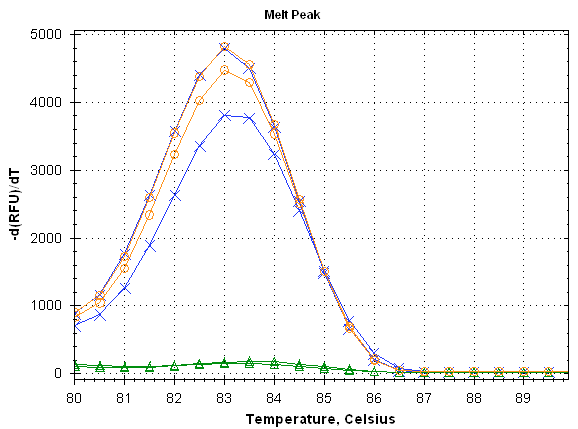


**Figure S1.** Melt curve analysis of RT-qPCR products.

Dissociation profiles of amplified products obtained by RT-qPCR, shown as the negative derivative of fluorescence with respect to temperature (−d(RFU)/dT). A major melting peak is observed around ~83°C for 6936 WT (orange circles) and *FPR1C* (blue crosses) strains, indicating specific amplification. In contrast, the *fpr1∆* strain (green triangles) shows no significant peak, consistent with the absence or strong reduction of the target transcript.


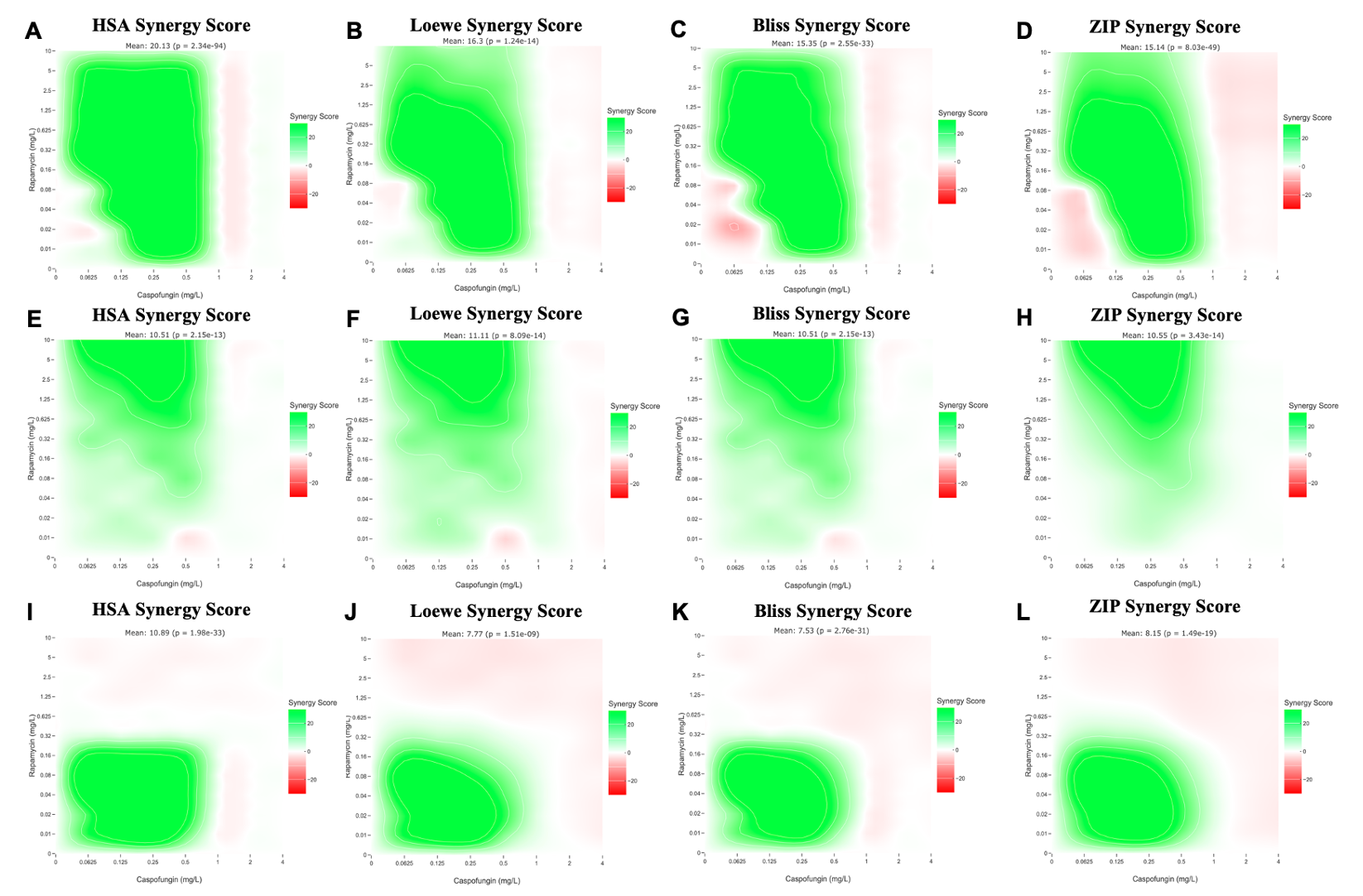


**Figure S2.** Synergy scores and matrices for caspofungin and rapamycin. The overall synergy scores as determined by the HSAana, Bliss, and ZIP models are shown. Each row represents a different *C*. *lusitaniae* strain: (**A**–**D**) 6936 WT strain, (**E**–**H**) *C*. *lusitaniae* *fpr1∆* strain, (**I**–**L**) C. *lusitaniae* *FPR1C* strain. The experimental data are expressed for each well as the percentage of growth in the presence of drugs relative to the growth control in drug‑free medium (Figure 2). These values were analysed using SynergyFinder 3.0 to generate 2D heatmaps and calculate synergy scores. In all heatmaps, green indicates synergistic interactions, whereas red denotes antagonistic interactions. *n* = 3.


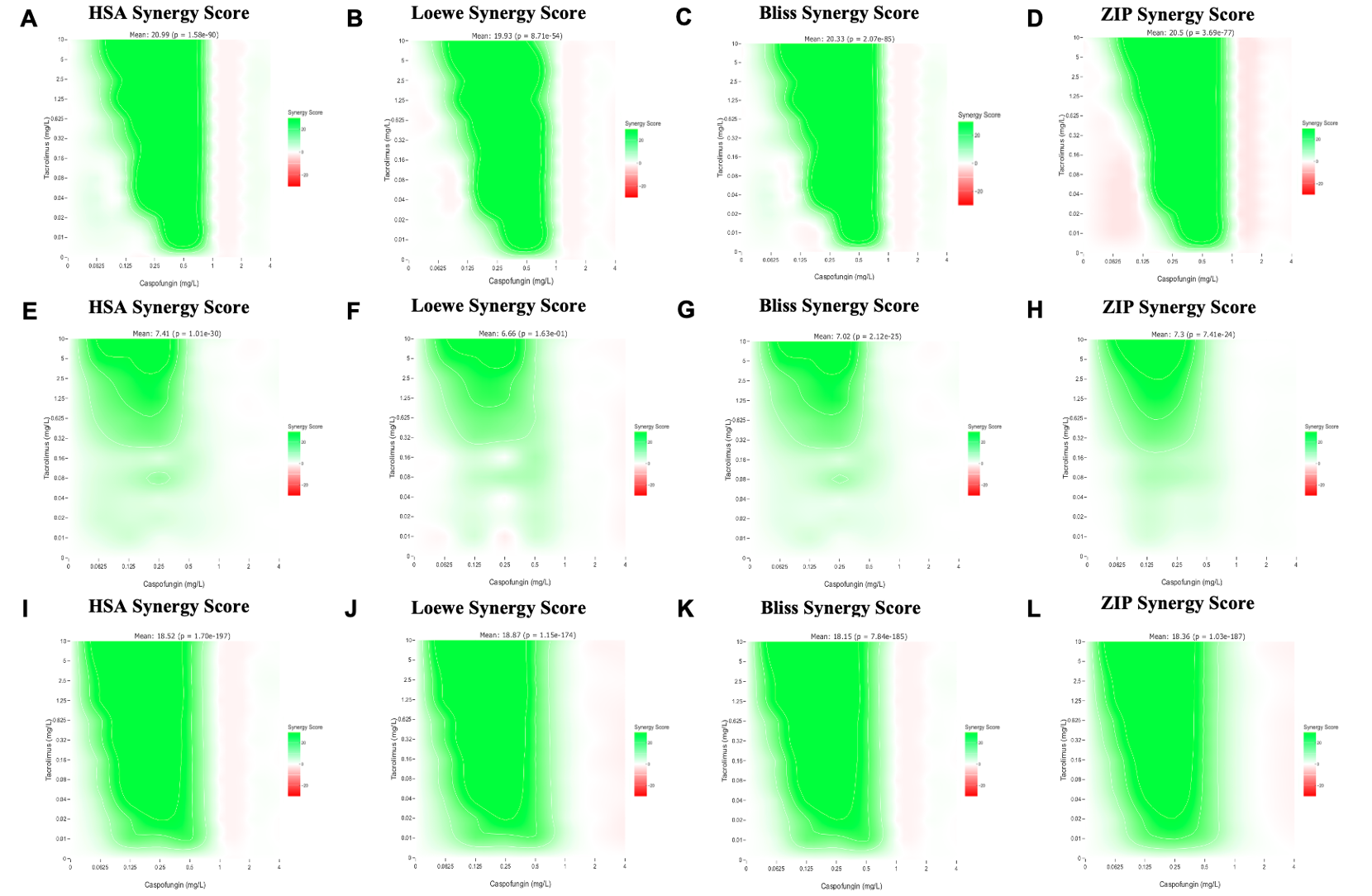


**Figure S3**. Synergy scores and matrices for caspofungin and tacrolimus. The overall synergy scores for the HSA, Loewe, Bliss, and ZIP models are shown. Each row represents a different *C*. *lusitaniae* strain: (**A**–**D**) 6936 WT strain, (**E**–**H**) *C*. *lusitaniae* *fpr1∆* strain, (**I**–**L**) *C. lusitaniae* *FPR1C* strain. The experimental data are expressed for each well as the percentage of growth in the presence of drugs relative to the growth control in drug‑free medium. These values were analysed using SynergyFinder 3.0 to generate 2D heatmaps and calculate synergy scores. In all heatmaps, green indicates synergistic interactions, whereas red denotes antagonistic interactions. *n* = 3.
